# The Common Variant rs4444235 near *BMP4* Confers Genetic Susceptibility of Colorectal Cancer: An Updated Meta-Analysis Based on a Comprehensive Statistical Strategy

**DOI:** 10.1371/journal.pone.0100133

**Published:** 2014-06-16

**Authors:** Li Liu, Qinji Su, Lixia Li, Xiaohui Lin, Yu Gan, Sidong Chen

**Affiliations:** 1 Guangdong Key Laboratory of Molecular Epidemiology and Department of Epidemiology and Biostatistics, School of Public Health, Guangdong Pharmaceutical University, Guangzhou, Guangdong, China; 2 Mental Health Center, the First Affiliated Hospital, Guangxi Medical University, Nanning, Guangxi, China; MOE Key Laboratory of Environment and Health, School of Public Health, Tongji Medical College, Huazhong University of Science and Technology, China

## Abstract

**Objective:**

We performed an updated meta-analysis, using a comprehensive strategy of a logistic regression and a model-free approach, to evaluate more precisely the role of the rs4444235 variant near the Bone morphogenetic protein-4 (*BMP4*) gene in susceptibility to colorectal cancer (CRC).

**Methods:**

A total of 19 studies with 28770 cases and 28234 controls were included. Metagen system with logistic regression was applied to choose the most plausible genetic model for rs4444235. Generalized odds ratio (OR_G_) metric was used to provide a global test of relationship between rs4444235 and CRC risk.

**Results:**

Metagen analysis suggested the rs4444235 fitted best to an additive model. In assessment of the additive model, heterogeneity was observed (*P* = 0.059, *I^2^* = 36.1), and pooled per-allele OR was 1.08 (95% CI = 1.05–1.11). Based on the model-free approach, pooled OR_G_ was 1.09 (95% CI = 1.05–1.14) under a random-effect model. Stratified analyses suggested heterogeneity could be in part explained by population ethnicity, study design, sources of controls, and sample size. Sensitivity analysis further supported the robust stability of the current results, by showing similar pooled estimates before and after sequential removal of each study.

**Conclusions:**

This meta-analysis provides a robust estimate of the positive association between the rs4444235 and CRC risk and further emphasizes the importance of the rs4444235 in CRC risk prediction.

## Introduction

Colorectal cancer is a major public health issue in developed countries and is becoming increasingly prevalent in Asia and Africa, with over 1.2 million new cases worldwide each year [1]. As other complex diseases, colorectal cancer is a complex trait driven by diverse etiologies involving in multiple environmental and genetic factors and their interactions [2]. Twin- and familial-based studies have provided clear evidence that approximately 35% of all CRC cases have a genetic component [2]. Of all CRC cases, <5% can be accounted by a combination of some germline mutations with high penetrance, whereas most “sporadic” cases are due to large numbers of common variants with individually small effects [3].

Recently, genome-wide association studies (GWAS) have implicated multiple common single nucleotide polymorphisms (SNPs) in inherited predisposition to CRC [4], [5]. The SNP rs4444235 at chromosome 14q22.2, mapping 9.4 kb upstream region of the gene encoding bone morphogenetic protein 4 (*BMP4)*, was firstly reported by a meta-analysis of GWAS data to be associated with CRC risk, with a combined OR of 1.11 (95% CI = 1.08–1.15, *P* = 8.1×10^−10^) [6]. BMP4 is an important member of the BMP signaling pathway, which involves in CRC development through regulation of colorectal stem cell differentiation [7]. This SNP has been proposed to act as a cis-regulator of *BMP4* and thus conferred to CRC risk [6]. However, the following replication studies yielded inconsistent results, in part due to “winner curse” in the original report [8], “Proteus phenomenon” in replication data [9], heterogeneous ethnical population, and insufficient statistical power, among other issues.

Meta-analysis, by integrating published data, may be a powerful tool to clarify the inconsistencies across individual studies. Two meta-analyses have been performed to assess rs4444235 in CRC. The meta-analysis by Li et al. [10], including 19893 cases and 22106 controls, assessed multiple genetic models for the rs4444235, which would lead to multiple comparisons or erroneous mode specification without priori biological evidence. The other meta-analysis by Theodoratou et al. [11], including less samples (18607 cases and 19576 controls), utilized a maximum likelihood estimator to decipher plausible model for the rs4444235. However, in this meta-analysis, there was no subgroup analysis undertaken. To overcome the above mentioned shortcomings in the previous meta-analyses, we integrated published data from 28770 cases and 28234 controls, and performed an updated meta-analysis, using a comprehensive statistical strategy. The methodology of logistic regression was applied to estimate the most plausible genetic model in the metagen system [12]. The generalized odds ratio, based on model-free approach, was utilized to provide a global test of genetic association [13]. Stratified analyses were further performed to explore potential sources of heterogeneity. The core aim of this meta-analysis was to provide a more precise and robust evaluation for the role of rs4444235 polymorphism in genetic susceptibility of colorectal cancer.

## Materials and Methods

### Search Strategy and Identification of Relevant Studies

This meta-analysis were conducted according to the Preferred Reporting Items for Systematic Reviews and Meta-analyses (PRISMA) statement (Checklist S1) [14]. Genetic association studies regarding rs4444235 and colorectal cancer (CRC) risk were searched in the PubMed/MEDLINE and EMBASE databases through October 15, 2013, by using the combinations of the keywords: (“BMP4” or “rs4444235” or “14q22.2”) and (“colorectal cancer” or “Colorectal neoplasmor” or “colon cancer” or “rectal cancer”). The similar search terms was also used for the WANFANG DATA and CNKI databases. The search was supplemented by review of reference lists for all relevant studies and review articles. All relevant reports identified were included without language restriction.

The following inclusion criteria should be fulfilled: (1) either case-control or nested case-control studies; (2) clear definition of colorectal cancer cases; (3) studies evaluating relationship between rs4444235 and CRC risk; (4) providing sufficient data to re-calculate the effect metrics, that was, numbers of genotypes in cases and controls. The authors were contacted via E-mail when eligible articles reported insufficient data. If they were unable to provide detailed data, those articles were excluded. Animal studies, reviews, conference abstracts, editorials and letters were excluded. If more than one ethnical population were in one report, each population was considered separately. Studies overlapping with other studies should be excluded, and the one with the most completed information was included. The first study on the association of rs4444235 by Houlston et al. was excluded [6], due to overlaps with the study by Tomlinson et al. [5]. The latter was chosen because of the larger sample.

### Data Extraction

Data were extracted independently and in duplicate by 2 reviewers (L. Liu & Q. Su). The following data was extracted from each article according to a fixed protocol: the first author, publication year, study design, country, ethnicity, source of controls, numbers of cases and controls, mean age of cases, sex ratio, site/type of colorectal cancer, genotyping method, minor allele frequency (MAF), and frequency of genotypes in cases and controls.

### Statistical Analysis

Hardy-Weinberg equilibrium in controls was re-analyzed using the goodness-of-fit χ^2^ test (*P*>0.05). The inverse variance method was applied to estimate the pooled frequency of the risk allele (the C allele) in various ethnical populations. The genetic effect of the rs4444235 in CRC susceptibility was assessed using the approaches described as below:

Metagen system has provided a general framework to decipher the most plausible genetic model for the rs4444235 that treated the genotypes as independent variables in a logistic regression under both fixed and random effects models [12]. Under fixed-effect model, two parameters, *θ*_2_ and *θ*_3_ were estimated using the logistic regression: logit (*π_ij_*) = *α_i_* +*θ_2_z_i2_*+*θ_3_z_i3_*, where *α_i_* was the indicator of study-specific fixed-effect, OR_TC/TT_ = exp(*θ_2_*), and OR_CC/TT_ = exp(*θ_3_*). In order to account for an additive component of heterogeneity, a random-effect logistic regression was performed using the GLLAMM module in STATA software via introducing a study-specific random coefficient: logit (*π_ij_*) = *α_i_* +(*θ_2_+ν_i2_*)*z_i2_*+(*θ_3+_ν_i3_)z_i3_*. The most plausible genetic model was determined using the following procedure: if *θ_2_* = *θ_3_* = 0, no significant genetic-association was suggested; if *θ_2_* = 0 and *θ_3_*>0, a recessive genetic model was suggested; if *θ_2_* = *θ_3_>*0, a dominant model was suggested; if *θ_3_*>*θ_2_>*0, a co-dominant model was suggested; if 2*θ_2_* = *θ_3_*, an additive model was likely. In this meta-analysis, the genetic model of rs4444235 was best fitted with an additive model. Then the per-allele OR of the C allele (additive model ) with corresponding 95% confidence interval (95% CI) was estimated in a logistic regression model, by assigning scores of 0, 1, and 2 to the AA, AC and CC genotypes, respectively. Between-study heterogeneity was assessed by the Cochran’s χ^2^ based *Q* test and *I^2^* metric. If there was no heterogeneity (i.e., if the *Q* test was significant [*P*<0.1] or *I^2^* was less than 25%), a fixed-effect model was used to pool the estimate; otherwise, a random-effect model was applied. To explore the sources of heterogeneity, stratified analyses were performed, if feasible, according to population ethnicity (Asians, Caucasians, and Africans), sources of controls (population- and hospital-based), study design (GWAS and replication study), and total sample size (≤2000 and >2000).

Additionally, the generalized OR (OR_G_), based on a genetic model-free approach, was also introduced in this meta-analysis [13]. The OR_G_ utilized the complete genotype distribution to provide an estimate of overall gene-disease relationship, given that the mutational load was treated as a graded exposure. Heterogeneity was also assessed for OR_G_ metric and stratified analysis was also performed.

Sensitivity analysis was performed to assess the influence of single study on pooled estimates. Publication bias was tested by the Egger’s regression test and Begg’s funnel plot. Statistical analyses were conducted in ORGGASMA, metan and metagen modules in STATA software version 13.0. A *P* value of <0.05 was considered statistically significant, except for estimation of between-study heterogeneity, where a significant level of 0.10 was applied.

## Results

### The Characteristic of Included Studies

**Figure 1:**
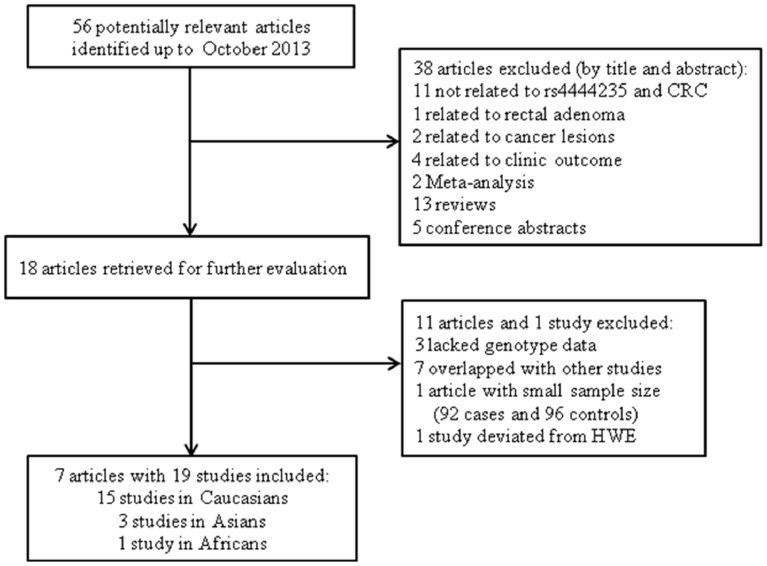
Flow chart of study selection process.

Figure 1 shows a flow diagram of the study selection process. The comprehensive search yielded 56 potentially relevant references. 18 articles were determined to be initially eligible by screening titles and abstracts. After further detailed evaluation, 7 duplicated articles [6], [15], [16], [17], [18], [19], [20] and 3 articles with insufficient data [20], [21], [22], [23] were excluded. 1 article was excluded due to small sample size (92 cases and 96 controls) [24]. 1 study in the article by Tomlinson et al. was excluded due to deviation with Hardy-Weinberg equilibrium [5]. Finally, a total of 7 articles with 19 studies of 28770 cases and 28234 controls were included in this meta-analysis [5], [25], [26], [27], [28], [29], [30]. The characteristics of these studies were summarized in Table 1. Among the included studies, 15 studies were performed in Caucasians, 3 studies in Asians, and 1 study in Africans.

**Table 1:** Characteristics of included studies in the meta-analysis of rs4444235 and colorectal cancer.

First author	Publication year	Ethnicity	Study design	Source of controls	MAF in controls	Cases			Controls		
						TT	TC	CC	TT	TC	CC
Fernandez-Rozadilla [25]	2010	Caucasian	Replication	Hospital	0.544	168	436	242	196	411	274
von Holst S [26]	2010	Caucasian	Replication	Population	0.439	573	829	356	533	838	326
Xiong F [27]	2010	Asian	Replication	Population	0.443	583	1091	427	639	1085	399
Kupfer SS [29]	2010	African	Replication	Hospital	0.334	332	319	62	400	418	97
Kupfer SS [29]	2010	Caucasian	Replication	Hospital	0.475	93	183	97	100	163	83
Ho JW [28]	2011	Asian	Replication	Hospital	0.522	170	350	195	168	346	199
Tomlinson (UK1) [5]	2011	Caucasian	GWAS	Population	0.452	233	441	247	274	470	184
Tomlinson (SCOT1) [5]	2011	Caucasian	GWAS	Population	0.451	256	500	220	294	512	195
Tomlinson (SCOT2) [5]	2011	Caucasian	GWAS	Population	0.451	540	1017	449	630	999	428
Tomlinson (VQ58) [5]	2011	Caucasian	GWAS	Population	0.468	503	886	410	773	1312	603
Tomlinson (CCFR) [5]	2011	Caucasian	GWAS	Population	0.476	290	595	298	274	496	227
Tomlinson (AU) [5]	2011	Caucasian	GWAS	Population	0.439	124	208	108	129	233	76
Tomlinson (HEL) [5]	2011	Caucasian	Replication	Population	0.426	272	459	202	273	405	150
Tomlinson (SEARCH) [5]	2011	Caucasian	Replication	Population	0.471	618	1083	537	650	1086	519
Tomlinson (COIN/NBS) [5]	2011	Caucasian	Replication	population	0.462	593	1044	510	722	1246	532
Tomlinson (UK3) [5]	2011	Caucasian	Replication	Population	0.472	2012	3865	1828	1247	2116	1006
Tomlinson (SCOT3) [5]	2011	Caucasian	Replication	Population	0.455	305	554	268	628	1130	432
Tomlinson (UK4) [5]	2011	Caucasian	Replication	Population	0.463	141	306	127	288	544	210
Li FX [30]	2012	Asian	Replication	Hospital	0.468	35	122	58	71	141	54

Abbreviations: GWAS, genome-wide association study; MAF, minor allele frequency (the C allele of rs4444235).

### Pooled Frequency of the Risk Allele (the C Allele) in Controls According to Ethnicity

Significant heterogeneity was seen both in Caucasians and Asians. and thus the random-effect model was applied (all *P*<0.0001, *I^2^* = 82.21 and 92.40, respectively). The pooled frequency of the C allele was 0.463 (95% CI = 0.452–0.474) in Caucasians, similar to that of 0.477 (95% CI = 0.423–0.532) in Asians. Only 1 study was conducted in Africans, and the frequency of the C allele was 0.334.

### Overall Meta-analysis of the rs4444235 and Colorectal Cancer Risk

**Figure 2:**
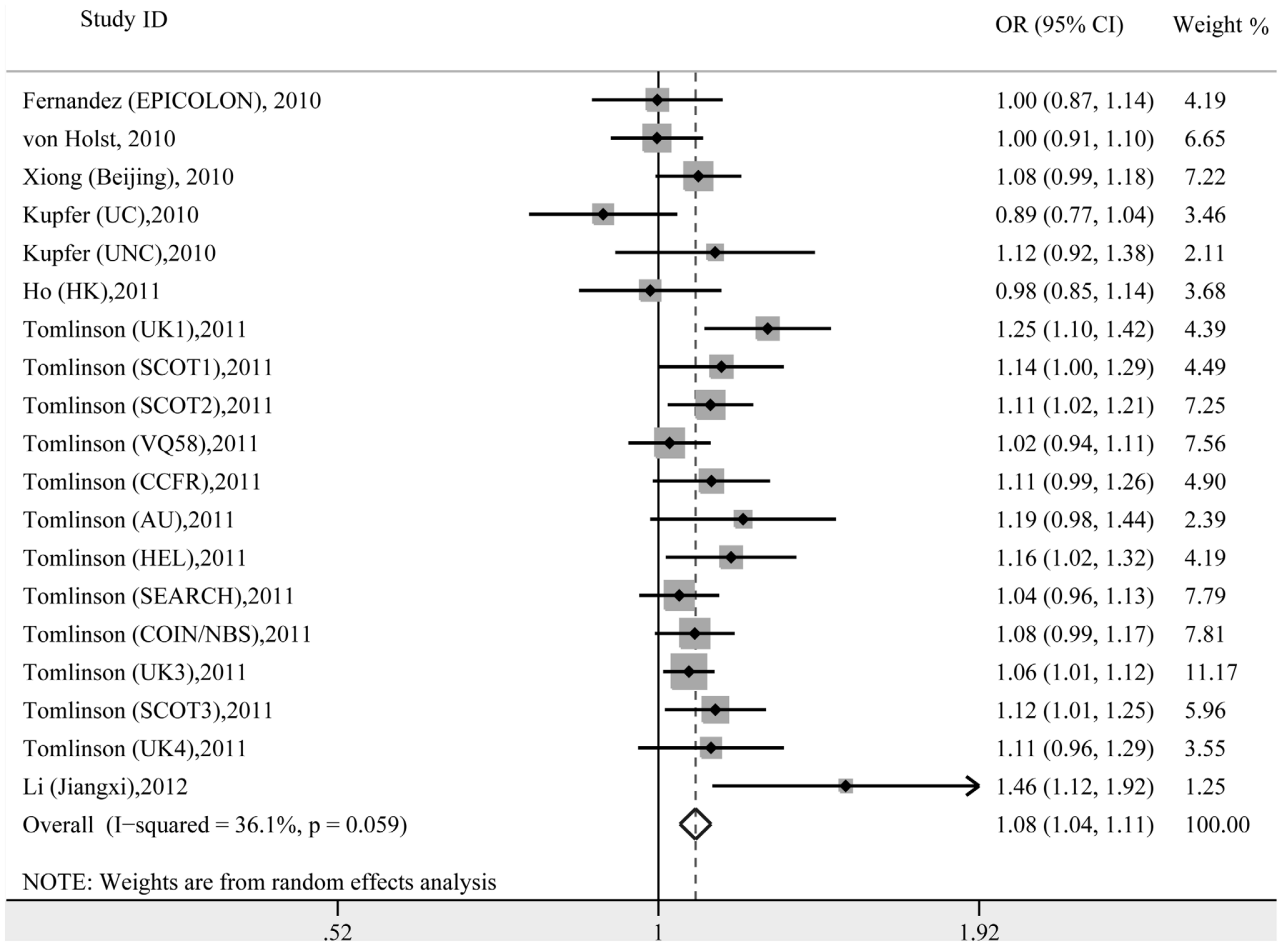
The forest plot of the association between rs4444235 and colorectal cancer risk in the additive model.

Table 2 summarizes the results of overall meta-analysis. In the metagen analysis, the pooled OR_TC/TT_ and OR_CC/TT_ were 1.08 (95% CI = 1.03–1.12) and 1.18 (95% CI = 1.12–1.25), respectively, suggesting an additive model as the most plausible genetic model. Then the additive model for the rs4444235 was assessed using traditional method. In the additive model, heterogeneity was observed (*P* = 0.059, *I^2^* = 36.1), and thus the random-effect model was applied. The variant was significantly associated with increased CRC risk, with a pooled per-allele OR of 1.08 (95% CI = 1.05–1.11; Figure 2). Based on the model-free approach, heterogeneity was also seen (*P* = 0.063, *I^2^* = 35.6). Under the random-effect model, significant result was also produced for the association of rs4444235 and CRC risk, with a pooled OR_G_ of 1.09 (95% CI = 1.05–1.14).

**Table 2:** Meta-analysis of rs4444235 and colorectal cancer risk.

Study characteristics		Cases/controls	Genetic model	OR (95%CI)	*I^2 (^*%)	*P* for heterogeneity
Total (N = 19)		28770/28234	Additive Model	1.08 (1.04–1.11)	36.1	0.059
			OR_G_	1.09 (1.05–1.14)	35.6	0.063
Ethnicity	Caucasian (N = 15)	25026/24217	Additive Model	1.08 (1.05–1.11)	11.8	0.321
	Asian (N = 3)	3031/3102	Additive Model	1.11 (0.95–1.31)	68.9	0.040
	African (N = 1)	713/915	Additive Model	0.89 (0.77–1.04)		
	Caucasian (N = 15)		OR_G_	1.10 (1.06–1.14)	11.7	0.322
	Asian (N = 3)		OR_G_	1.14 (0.94–1.37)	68.8	0.041
	African (N = 1)		OR_G_	0.88 (0.74–1.05)		
Sources of controls	Population based (N = 13)	23807/22990	Additive Model	1.08 (1.05–1.12)	16.6	0.277
	Hospital based (N = 6)	4963/5244	Additive Model	1.05 (0.95–1.15)	59.5	0.030
	Population based (N = 13)		OR_G_	1.10 (1.06–1.14)	15.3	0.290
	Hospital based (N = 6)		OR_G_	1.06 (0.94–1.18)	59.3	0.031
Study design	GWAS (N = 6)	7325/8109	Additive model	1.12 (1.06–1.18)	33.4	0.185
	Replication (N = 13)	21445/20125	Additive model	1.06 (1.05–1.11)	33.6	0.114
	GWAS (N = 6)		OR_G_	1.14 (1.07–1.22)	31.0	0.203
	Replication (N = 13)		OR_G_	1.07 (1.03–1.12)	33.3	0.116
Total sample size	≤2000 (N = 10)	6706/7358	Additive model	1.11 (1.03–1.19)	57.6	0.012
	>20000 (N = 9)	22064/20876	Additive model	1.07 (1.05–1.11)	0.0	0.674
	≤2000 (N = 10)		OR_G_	1.13 (1.03–1.23)	57.1	0.013
	>20000 (N = 9)		OR_G_	1.08 (1.05–1.12)	0.0	0.666

Abbreviations: GWAS, genome-wide association study; OR, odds ratio; 95% CI, 95% confidence interval; OR_G,_ generalized OR.

### Stratification Analysis of the rs4444235 and Colorectal Cancer Risk

When performed stratified analysis by population ethnicity, in Caucasian subgroup of 15 studies, heterogeneity was removed, and the significant association of the rs4444235 still existed for both additive model and OR_G_ assessment (Table 2). However, in Asians of 3 studies, there was significant heterogeneity (*P* = 0.040 and 0.041 for additive model and OR_G_, respectively), and no significant association was found.

According to the sources of controls, in the population-based subgroup of 13 studies, analysis of the additive model and OR_G_ both showed significant association of rs4444235 with CRC without evidence of heterogeneity, whereas in the hospital-based subgroup of 8 studies, significant heterogeneity was observed and no significant association was reported.

Regarding to study design, there were 6 GWAS and 13 replication studies. When assessing the additive model and OR_G_ metric, both subgroups showed the positive genetic association with CRC risk, without evidence of heterogeneity. Interestingly, the pooled estimates in the GWAS (per-allele OR = 1.12; OR_G_ = 1.14) were slightly larger than those in the subgroup of replication studies (per-allele OR = 1.06; OR_G_ = 1.07).

The stratified analysis was also conducted according to total sample size (numbers of both cases and controls), into 2 subgroups: the large sample size subgroup (total sample size >2000) with 22064 cases and 20876 controls and the small or moderate size subgroup (total sample size ≤2000) with 6706 cases and 7358 controls. For both additive model and OR_G_ analyses, heterogeneity was removed in the subgroup with large sample size, whereas in the small or moderate size subgroup, heterogeneity still existed. Both subgroups showed the significant association between the rs4444235 and CRC risk.

### Sensitivity Analysis and Publication Bias Assessment

Since between-study heterogeneity was observed in this meta-analysis, we further performed sensitivity analysis under the random-effect model. For the additive model, the sensitivity analysis, by sequentially omitting each study, reported a series of pooled OR with 95% CI exceeding 1.00, and the pooled ORs were similar before and after omitting each study (Table 3). Similar results were suggested for OR_G_ analysis that no single study significantly altered the pooled OR_G_. In the Begg’s and the Egger’s tests, there was no evidence of publication bias for both additive model and OR_G_ (all *P* values for Begg’s and Egger’s tests >0.05).

**Table 3:** Sensitivity analysis of rs4444235 and colorectal cancer risk.

Omitted study	Additive model			OR_G_		
	OR (95% CI)	*P* *	*I^2^*	OR (95% CI)	*P* *	*I^2^*
Fernandez (EPICOLON) [25]	1.08 (1.05–1.12)	0.059	37.0	1.10 (1.06–1.14)	0.065	35.9
von Holst [26]	1.08 (1.05–1.12)	0.082	33.6	1.10 (1.06–1.14)	0.091	32.4
Xiong (Beijing) [27]	1.08 (1.04–1.12)	0.043	39.6	1.09 (1.05–1.14)	0.046	39.0
Kupfer (UC) [29]	1.08 (1.05–1.12)	0.173	23.8	1.10 (1.06–1.14)	0.174	23.7
Kupfer (UNC) [29]	1.08 (1.04–1.11)	0.045	39.3	1.09 (1.05–1.14)	0.048	38.7
Ho (HK) [28]	1.08 (1.05–1.12)	0.062	36.4	1.10 (1.06–1.14)	0.066	35.8
Tomlinson (UK1) [5]	1.07 (1.04–1.10)	0.159	25.2	1.08 (1.05–1.12)	0.164	24.6
Tomlinson (SCOT1) [5]	1.08 (1.04–1.11)	0.053	37.9	1.09 (1.05–1.13)	0.056	37.4
Tomlinson (SCOT2) [5]	1.08 (1.04–1.11)	0.051	38.3	1.09 (1.05–1.14)	0.055	37.5
Tomlinson (VQ58) [5]	1.08 (1.05–1.12)	0.062	36.5	1.10 (1.06–1.14)	0.065	36.0
Tomlinson (CCFR) [5]	1.08 (1.04–1.11)	0.047	38.9	1.09 (1.05–1.14)	0.051	38.3
Tomlinson (AU) [5]	1.08 (1.04–1.11)	0.057	37.2	1.09 (1.05–1.13)	0.058	37.0
Tomlinson (HEL) [5]	1.08 (1.04–1.11)	0.060	36.7	1.09 (1.05–1.13)	0.064	36.2
Tomlinson (SEARCH) [5]	1.08 (1.05–1.12)	0.049	38.5	1.10 (1.05–1.14)	0.052	38.0
Tomlinson (COIN/NBS) [5]	1.08 (1.04–1.12)	0.043	39.7	1.09 (1.05–1.14)	0.046	39.1
Tomlinson (UK3) [5]	1.08 (1.04–1.12)	0.045	39.3	1.10 (1.05–1.14)	0.047	38.9
Tomlinson (SCOT3) [5]	1.08 (1.04–1.11)	0.052	38.0	1.09 (1.05–1.14)	0.054	37.7
Tomlinson (UK4) [5]	1.08 (1.04–1.11)	0.045	39.2	1.09 (1.05–1.14)	0.049	38.6
Li (Jiangxi) [30]	1.07 (1.04–1.11)	0.145	26.5	1.09 (1.05–1.13)	0.153	25.7

Abbreviations: OR, odds ratio; 95% CI, 95% confidence interval; OR_G,_ generalized OR.

*P values for heterogeneity were calculated by the Cochran’s χ^2^ based Q test.

## Discussion

Currently, traditional meta-analyses of genetic association studies are usually performed by collapsing genotypes in two categories assuming various genetic models. However, these different models are not independent, and a priori biological justification for the choice of a specific model is seldom available [31]. Additionally, interpretation of these results is complicated since a set of different estimates and significance tests are usually provided. In this current meta-analysis of rs4444235 and colorectal cancer risk, we utilized a comprehensive strategy, including the metagen analysis based on logistic regression and OR_G_ metric based on model-free approach [12], [13], to overcome the drawbacks in traditional meta-analysis of erroneous model specification and multiple model tests with an inflated Type I error rate, and make the interpretation of the current results easier.

In this meta-analysis of 19 case-control studies of 28770 cases and 28234 controls, the metagen analysis indicated that the rs4444235 fitted best to an additive model. Knowledge of the best-fitting model for the rs4444235 may be important in optimizing the use of this SNP in colorectal cancer (CRC) risk prediction. Assessment of additive model indicated that CRC risk was increased by 8% per extra C allele. Based on model-free approach, the generalized OR (OR_G_) analysis showed that CRC cases with higher mutational load than healthy individuals have 9% higher risk for CRC susceptibility. Sensitivity analysis further supported the current results, by showing similar ORs before and after sequentially omitting single study. The positive association of the rs4444235 with CRC risk identified by this meta-analysis was also concordant with the findings of previous meta-analyses [10], [11].

rs4444235 is 9.4 kb from the transcription start site of the *BMP4*. The BMP signaling has vital function in maintenance of Wnt signaling to inhibit differentiation of stem cell near colorectal crypt bases [7]. Heightened expression of BMP pathway members would restrain the Wnt signaling, subsequently activate β-catenin and elevate cells susceptibility to tumor-causing mutations, and ultimately promote colorectal carcinogenesis [7]. Intriguingly, in a recent study, luciferase reporter assay suggested the element to which rs4444235 maps acts as an allele-specific transcriptional enhancer [23]. In CRC cell lines allele-specific expression analysis indicated a significant association of increased *BMP4* expression with the C allele [23]. These data have strongly supported the functional role of rs4444235 in CRC development through the cis-acting regulatory influence on *BMP4* expression.

Heterogeneity is a pervasive and difficult problem in meta-analysis of genetic association studies. Not surprisingly, heterogeneity existed in this meta-analysis, and thus the findings should be interpreted with caution. Nevertheless, in stratified analysis by ethnicity, heterogeneity was removed in Caucasians and significant association of rs4444235 retained. According to study design, both in GWAS and replication studies, heterogeneity was effectively decreased, and association was also existed. Interestingly, the subgroup of GWAS yielded larger pooled ORs than that in replication data, indicating “winner curse” existed for the rs4444235 in GWAS. In regarding to sample size, only in the subgroup with large sample size heterogeneity was removed, but both subgroups showed significant genetic association. When stratified by sources of controls, heterogeneity was removed in population-based subgroup. These findings suggested the heterogeneity could be in part explained by the distinct natures of population ethnicity, control sources, study design, and sample size across individual studies. Furthermore, no single study had significant influence on the overall estimates in sensitivity analysis, and no publication bias was observed in this meta-analysis, suggesting the robust stability of the current results.

Despite the strength of this study utilizing a comprehensive statistical strategy, some limitations merit serious consideration. In stratified analysis by ethnicity, majority of studies were conducted in Caucasians, only 3 studies and 1 study appraised rs4444235 in Asians and Africans, respectively. No association was seen in Asians and Africans possibly due to small sample size and insufficient power. The relationship of rs4444235 and CRC risk merits more studies in various populations. Only one polymorphism was assessed in this meta-analysis, and this meta-analysis did not give a global view of the genetic variants of *BMP4* in CRC susceptibility. Additionally, gene-environment interactions did play more important role in colorectal carcinogenesis as compared with genetic factors [32]. However, only one study so far by Hutter et al. has explored interaction of rs4444235 and environmental factors [33], and thus the interaction could not be appraised in this meta-analysis.

In conclusion, this updated meta-analysis, utilizing a comprehensive strategy, further supports the significant role of rs4444235 in genetic susceptibility of colorectal cancer. Further functional polymorphism-based studies in the whole *BMP4* gene are warranted to confirm and extend the current findings in various ethnical populations.

## Supporting Information

Checklist S1 **Checklist of Preferred Reporting Items for Systematic Reviews and Meta-analyses statement.**
(DOC)
